# Active Food Packaging Based on Biopolymers and Aroma Compounds: How to Design and Control the Release

**DOI:** 10.3389/fchem.2019.00398

**Published:** 2019-06-04

**Authors:** Jose Daniel Wicochea-Rodríguez, Pascale Chalier, Thierry Ruiz, Emmanuelle Gastaldi

**Affiliations:** UMR 1208 Ingénierie des Agropolymères et Technologies Emergentes, Université de Montpellier-SupAgro-INRA-CIRAD, Montpellier, France

**Keywords:** aroma, clay, cyclodextrin, biopolymer, interaction, retention, release

## Abstract

Aroma compounds are known to be efficient active agents for a broad range of applications (antimicrobial, anti-oxidant, insect repellent…) that are highly sought when aiming at extending shelf life of food or biological products. However, they are intrinsically odorant and volatile at ambient temperature, which restricts the processing routes used to introduce them in a polymeric matrix and can affect their mode of action and limit efficiency. Indeed, due to their high sensitivity toward temperature they can be lost or transformed during processing. Acting after being released in the headspace, their concentration has to be controlled to avoid any odorant contamination of the targeted products. Hence, the ability for an aroma compound to be retained in a polymeric matrix, and then released when submitted to a triggering effect, are the two main requirements that should be satisfied. The volatile nature of the aroma compound offer the possibility when introduce in the packaging to act by direct or indirect contact with the product and thus to be used in different ways; as a coating layer directly applied on the product surface, as a self-supported film or as coated paper when associated with a paper sheet, as well as an object that could be inserted in the package. As biopolymers such as proteins and polysaccharides are able to retain aroma compounds but also to favor their release by modification of their structure when the relative humidity (RH) and temperature change, they are relevant carriers of these specific aroma compounds. Examples of how active packaging systems with limonene, eugenol and carvacrol as active agents were designed and elaborated. These examples will be presented with a special focus on the processing conditions and the way to improve their aroma compound retention and the release control (biopolymer nature, cyclodextrin clay addition…). Avrami's equation has been used to model the transfer of aroma compound and to advantageously compare it taking into account the mechanism in relation to the biopolymer structural changes.

## Introduction

In a context of growing demand for food that is fresh or minimally processed, free of synthetic preservatives and at the same time having an extended shelf life, the developing of efficient packaging technologies has appeared as an inescapable need (Vermeiren et al., 1999; Ramos et al., 2012; Efrati et al., 2014). To face this challenge, active packaging have been developed to avoid the current degradation reactions of foodstuffs incorporating deliberately components that would release or absorb substances present in the packaged food or into the packaging headspace (Labuza and Breene, 1989; Vermeiren et al., 1999; Han, 2005; Malhotra et al., 2015). Among the components, which can be incorporated into the packaging matrix, antimicrobials from synthetic or natural origin are provided to prevent food spoilage caused by microbial growth or undesirable chemical changes due to oxidation. As consumers have become more aware about potential health issues associated with synthetic preservatives and focused on ingredients from natural sources, the use of natural food additives in active packaging has raised growing interest for improving the shelf life of perishable products (Baldwin et al., 2011; Efrati et al., 2014).

Essential oils (EOs) and their main aroma compounds are known for their extensive properties and among them antimicrobial and anti-oxidant activity (Alma et al., 2007; Mkaddem et al., 2009; Alves-Silva et al., 2013; Rivera et al., 2015). Because of their natural origin, the use of EOs or selected aroma compounds as additives in food packaging is a convenient alternative (López et al., 2007; Rodríguez-Lafuente et al., 2007). Moreover, due to their volatile nature, they could be released from the packaging material as a vapor to the headspace without necessity to have an intensive contact between the food product and the packaging material. By consequence, they can easily diffuse toward the food surface where microbial growth and spoilage occur. However, the release of aroma compounds from the packaging material need to be controlled in such a way to limit their concentration and increase their efficiency. For this purpose, EOs or an aroma compound could be trapped in carrier-biopolymer matrix (Campos et al., 2011).

Biopolymers stemming from the waste streams generated during agro-industrial processing have emerged as suitable candidates for playing the role of potential carrier. Indeed, they present the double benefit to limit the presence of synthetic additives in food and to promote the valorization of sustainable raw materials instead of conventional plastics, whose accumulation in the environment raises serious environmental concerns. Combining EOs with such biopolymers is believed to induce the retention of aroma compounds by establishing specific molecular interactions.

Among biopolymers, polysaccharides and proteins are often used for encapsulation of EOs since they have shown both good retention and release properties, and moreover they are available at relatively low cost (Reineccius, 1989; Kim and Morr, 1996; Madene et al., 2006). They are also used as edible films and coating in regard to their good film forming properties, oxygen permeability (OP) similar to that of plastic films. Then, active packaging based on soy protein, gluten, whey protein but also chitosan, starch, pectins, alginates, and arabic gum have intensively been designed and tested against a wide range of micro-organisms (Pavlath et al., 1999; Ben Arfa et al., 2007b; Ali et al., 2010; Balaguer et al., 2013; Pérez Espitia et al., 2014; Barba et al., 2015; Antosik et al., 2017). However, the major drawback of these films is their higher water vapor permeability (WVP) due to the hydrophilic nature of biopolymers (Gennadios et al., 1994). To overcome this limitation, biopolymers are often combined with nanofillers (as montmorillonites nanoclays) known to impart convenient barrier properties (OP and WVP) to the resulting films through the achievement of a nanocomposite structure (Perisco et al., 2009; Mascheroni et al., 2011;Qi et al., 2016).

Carvacrol, eugenol, and limonene are three aroma compounds present in several EOs and having recognized antimicrobial properties (Di Pasqua et al., 2007; Devi et al., 2010). However, they exhibit contrasted properties in terms of polarity and volatility. Therefore, the retention and release properties of each one of these three compounds will strongly depend on the matrix carrier (polymer) and their interactions with it and the environment, as well as the film preparation method (Mastromatteo et al., 2010; Ribeiro-Santos et al., 2016).

A better knowledge of the matrix interactions with aroma compounds can be helpful to predict their mobility once introduced in the polymer matrix, which is a key element in understanding the mechanisms of release in the headspace or in contact with food. Therefore, controlling both the retention and the release of aroma compounds is a challenging requirement when aiming at designing and processing an efficient active packaging. Among the parameters involved in the preparation of such packaging materials, conditions and matrix formulation (e.g., polymer nature and concentration, addition of plasticizers, emulsifiers, fillers) together with the drying conditions are expected to influence the retention and thus the release of the active compound (Kurek et al., 2012). In consequence, designing an efficient antimicrobial packaging not only depends on the specificity of the active agent that should be chosen according to the type of food targeted and the nature of deteriorative microbial flora, but also on the kinetic of release of the active agent.

The present study investigated first, how the formulation of packaging can influence the retention of aroma compounds when designing active packaging. For this purpose, the focus will be put on the choice of (i) the biopolymeric carrier/matrix; soy protein isolates (SPI), pectins, arabic gum (AG), and wheat gluten (WG), (ii) the nature and concentration of aroma compound; limonene, carvacrol, and eugenol, (iii) the introduction of cyclodextrins and montmorillonites in the packaging film, and (iv) the fact that the film was self-supported or coated on paper. In a second part, the way to control the release will be investigated. The transfer of aroma compound will be modeled by Avrami's equation allowing comparing the release mechanism and transfer rate of the different aroma compounds taking into account the biopolymer structural changes.

## Materials and Methods

### Materials and Reagents

#### Materials

A commercial base paper (70 g/m^2^) was provided by Ahlstrom Research and Services and was used as support for coating.

Sodium montmorillonites, MMTs (Nanofil EXM757) were purchased from Sud Chemie AG (Germany). Soy protein Isolate (SPI) was supplied by Seah International (SAMPROSOY 90 NB, Wimille, France) contains 91.8% proteins and 8% moisture content. Vital wheat gluten,WG (12% moisture wet mass basis) was supplied by Amylum (Aalst, Belgium). High methoxy pectin powder (HMP: DE > 50) stemming from apple was purchased from (Sigma Aldrich Germany). Acacia *senegal* gum was provided by Alland and Robert Company natural and organic gum (Port Mort, France).

#### Chemicals

Glycerol was purchased from Acros organics (Ilkirch, France), acetic acid, sodium sulphite, and sorbitan monolaurate from Fluka-Sigma, Aldrich (Saint Quentin Fallavier, France). The antimicrobial agents, eugenol, limonene and carvacrol, as well as 2-heptanol and 2-nonanol used as internal standard were purchased from Sigma Fluka-Aldrich (Saint Quentin Fallavier, France). *n*-pentane (purity 95%) used as extraction solvent was furnished by VWR chemicals (France). Inclusion complex of carvacrol made with β-cyclodextrins and having a carvacrol content of 14.7% was supplied by Mane (Bar le Loup, France).

### Film and Coating-Forming Solutions

#### Soy Protein Isolate (SPI)

SPI was used to prepare films or coated papers. SPI suspensions at 10% w/w were prepared by dispersing 10 g of SPI powder in 80 mL of distilled water previously heated at 50°C under continuous magnetic stirring at 500 rpm for 30 min. Then, glycerol (2 g) were added as a plasticizer and the solution was stirred again for 30 min under the same conditions. This step was suppressed in the formulation of coating solution for paper. Then, the suspension was cooled to 25°C and a given amount of aroma compound (1.5 and 3 g for carvacrol and eugenol against 0.5–1 g for limonene) was added and homogenized at 8,000 rpm for 20 min by using high shear lab mixing (Silverson L4RT, England). Next, an ultrasonic treatment was applied for 15 min to eliminate air bubbles by using a (Qsonica Q700, USA). Depending on the amount introduced in the suspension, the final concentration of aroma compound in the film or coating was 5, 10, 15, or 30% w/w related to dry SPI.

β-cyclodextrins (CD) were added to the film-forming solution to obtain a coated paper with carvacrol/CD inclusion complex. In the presence of CD, the SPI content was limited to 5% w/w and the homogenization step was ensured by magnetic stirring (30 min at 500 rpm) to prevent the destruction of inclusion complexes and the release of aroma compound in the dispersion.

#### Acacia Gum (AG)

Acacia gum film-forming solution (20% w/w) was prepared by dispersing 20 g of AG in 90 mL of acetate buffer at 10 mM and pH = 5 heated at 40°C and by stirring at 500 rpm for 30 min. The suspension was cooled to room temperature and mixed at 500 rpm using magnetic stirrer overnight to ensure a complete dispersion of the gum. The pH of the suspension was adjusted at 5.6 using NaOH 1M after addition of glycerol (2 g), limonene (1 g) was added to the suspension before mixing at 8,000 rpm for 20 min using high shear lab mixing (Silverson L4RT, England). Finally, the suspension weight was adjusted by adding distilled water to obtain a 20% w/w suspension.

#### Pectin

Pectin suspensions (1, 2, and 3% w/w) were prepared by dispersing 1, 2, and 3 g of high methoxyl pectin in 90 mL of distilled water and heated to 90°C under continuous magnetic stirring at 500 rpm for 1 h. The solution was then cooled to room temperature before addition of glycerol (0.2 g) and mixed for 30 min. Limonene (1 g) was added to each suspension in the presence of sorbitan monolaurate as an emulsifier to favor the dispersion of the aroma compound. Then, the suspensions were mixed at 8,000 rpm for 20 min using a high shear lab mixer. The weight of each pectin suspension were finally adjusted by adding distilled water to obtain 1, 2, and 3% w/w suspensions.

#### Wheat Gluten

Wheat gluten (WG) powder 20 g was dispersed in 50 mL of distilled water containing 0.04 g of sodium sulfite as reducing agent. Pure acetic acid was added to the mixture under magnetic stirring to adjust the pH value of the solution below 4. Glycerol (2 g) was added to the WG suspension, which was mixed under high shear lab mixing at 5,000 rpm for 10 min. The suspension was put under vacuum in order to be degassed. Then, 3 or 6 g of carvacrol (i.e., carvacrol/WG = 15 or 30% w/w db gluten) was added to the suspension, which was mixed again under high shear lab at 5,000 rpm for 10 min (Silverson L4RT, England). Finally, the weight of the WG suspension was finally adjusted by adding distilled water to obtain a 20% w/w solution.

#### Nanoclays Addition in the Film and Coating-Forming Solutions

MMT were introduced in the coating-forming solutions as nanoclays suspension before the addition of aroma compound during the preparation of the solution. The nanoclays suspension was prepared in a preliminary step by dispersing a given amount of MMT powder into 30 mL of distilled water under magnetic stirring at 500 rpm for 12 h. Whatever the MMT content, 2 g of glycerol were added to the clay suspension under high shear lab mixing (Silverson L4RT, England) at 5,000 rpm for 15 min followed by an ultrasonic treatment applied for 15 min to the MMT-glycerol mixture. In a second step, the biopolymer solution and the MMT-glycerol suspension at the desired amount were mixed together under high shear lab mixing at 5,000 rpm for 10 min. In the last step, defined amounts of aroma compound (15 or 30% w/w db) were added to the solution as described above. The final weight of the suspensions was adjusted by adding distilled water to obtain 10 and 20% w/w SPI and WG suspensions, respectively.

#### Viscosity of the Film and Coating-Forming Solutions

The rheological behavior of solutions was determined at 25°C using an MC1 rheometer (Physica Rheolab, Anton Paar, Germany) equipped with a MSZ1 concentric cylinder (DIN/double gap measure cell). The consistency index, K in Pa.s and n, the flow behavior index were determined according to the Ostwald-de Waele rheological model using the Equation 1:

(1) $$\begin{array}{r} \sigma =\text{K}\gamma^{\text{n}} \end{array}$$

where σ is the shear stress applied (Pa) and γ the shear rate (s^−1^). A speed ramp was chosen at increasing shear rates from 50 to 1,250 s^−1^ using an equilibrium time of 120 s. All measurements were done in triplicate.

### Preparation of Films and Coatings

#### Films Process

Biopolymer-based films were prepared by casting method consisting in pouring a specified volume of film forming solution into glass Petri dishes (90 mm in diameter). Then the films were dried for 4–24 h in an oven set at 50 or 25°C and at a relative humidity varying between 40 and 50%. The volume and the % of dry matter of the initial solution were used to pre-determine the thickness of the final films.

#### Coating Process on Paper

The process used for coating paper consisted in spreading the film-forming solution over a paper sheet using an adjustable micrometer thin-layer chromatography applicator (Braive Instrument, Chécy, France). The paper sheet was previously wetted with water to avoid the wrinkling of paper and was maintained on an iron-perforated plate under partial vacuum (21 × 30 cm). The coating process was performed at 25°C, and depending on the matrix, aroma compounds and the final thickness wanted, the coated papers were dried for 3 up to 16 h at 23 ± 2°C and 50 ± 5% of RH.

### X-Ray Diffraction of WG/MMT/Carvacrol Films

Wide angle X-Ray diffraction (XRD) measurements were performed on MMT powder alone or mixed with eugenol or carvacrol using a Philips X'Pert MPD diffractometer (θ-2θ), working in reflection geometry and equipped with the X'celerator detector using the Cu-Ka radiation (λ = 1.5418 Å) generated at 40 kV/20 mA and a nickel filter. Data gathering was performed in continuous mode for θ angles from 2 to 30° with a scan rate of 2°min^−1^. Peak detection and analysis were obtained by means of the X'pert Highscore software without baseline subtraction.

### Retention and Release of Aroma Compound From Biopolymer Films and Coatings

#### Extraction Aroma Compounds and GC Analysis

The extraction procedure consisted to immerse a piece of films or coated paper, previously weighted, in 10 ml mixture of water and *n*-pentane (1:1) for SPI, AG, and pectins matrix or in 10 mL of n-pentane when WG is used as matrix. The extraction solvents were selected in function of the matrix solubility and the aroma compounds as previously described (Ben Arfa et al., 2007b; Mascheroni et al., 2011). Whatever the material, 100 μL of 2-nonanol or 2-heptanol (10 mg/mL in ethanol) as internal standards were added in the solution and the mixture was shaken for 16–24 h under magnetic agitation (500 min^−1^) at room temperature. The organic phase containing aroma compounds and internal standard was removed, dried over ammonium sulfate and then analyzed by gas chromatography. The analysis was carried out on a Varian 3800 GC-FID (Les Ulis, France) equipped with a DB5 column (J&W scientific) (30 m × 0.32 mm, film thickness 0.25 μm) and a flame ionization detector (FID; hydrogen: 30 mL/min; air: 300 mL/min; nitrogen: 30 mL/min). Hydrogen was used as carrier gas with a flow rate of 1.5 mL/min. The oven temperature was programmed to rise from 60 to 150°C at 6°C/min, then at 15°C/min to 250°C and held at 250 °C for 10 min. Injector and detector temperatures were adjusted at 250 and 300°C, respectively. Injections were done in split mode with a 1:20 ratio.

The extractions and GC analysis were done in triplicate. In order to quantify the aroma compound amount, an external calibration was carried out using five solutions of the aroma compound and the internal standard in the range of 10 and 1,000 ppm. The response coefficients relative to the internal standard for each aroma compound were determined and applied in calculation.

#### Retention of Aroma Compounds in Films and Coated Papers

Prior determining the aroma content remaining in films and coatings after drying, extraction yields were estimated for each material by depositing a known quantity of aroma compound on films and by applying the extraction procedure described above. The extraction yield varied between 85 and 100% depending on the aroma compound and matrix.

The % of retention of aroma compound after the casting and drying process was calculated by comparing the residual amount in films and the theoretical content of the aroma compound introduced in the material. The residual amount (g.g^−1^) was reported to the dry matrix content of the film calculated taking into account the glycerol, the moisture and aroma compound content of the film as described in the following equation:

Residual amount g.g^−1^ of dry matrix content = Residual amount g.g^−1^ of film × (1- B)

with B = moisture content-glycerol-aroma compound.

#### Kinetic Release During Storage in Controlled (RH% and T°C) Conditions

Pieces of films or coated papers of a determined surface (between 2.25 and 9 cm^2^) were weighted and put in a conditioning chamber (about 370 cm^3^) where the conditions of temperature and relative humidity were controlled. To adjust the relative humidity at the selected values, saturated salts solutions were added in the chamber and a humidified air was also fluxed in it. Air flow and temperature difference between the chamber and the environment were adjusted as previously described (Chalier et al., 2009; Mascheroni et al., 2011; Nassar et al., 2017).

Material samples were then taken off the oven at prescribed time intervals and extracted by the procedure described above to quantify the aroma compound content by gas chromatography analysis. Results were expressed in terms of remaining aroma compound at the time (*t*) per gram of films or coated papers and compared to the content at time t_0_.

## Results

### Influence of the Formulation of Active Films and Coatings on the Retention of Aroma Compounds

Different matrix formulation in terms of nature of biopolymer, concentration, suitable solvent system, addition of plasticizers, or fillers have been set up to prepare filmogenic solutions in which aroma compound have been introduced. Those solutions have then been deposited on different surfaces to produce films, coated-paper, and coated-fruit. The retention of active compound by those different materials has been compared to better understand how improving this key parameter. Indeed, it is a discriminant factor when antimicrobial films are designed with aroma compounds where only a sufficient amount needs to be released to act against microorganisms. Furthermore, it is also necessary to limit the inevitable losses of active agent occurring during the process due to the volatility of aroma compounds, and are unfavorable regarding cost and environmental impact.

#### Selection of the Polymer Carrier and Suitable Conditions of Dispersion for a Given Aroma Compound

Biopolymer carriers are expected to induce the retention of aroma compound through the implementation of specific molecular interactions. Such interactions strongly depend on the nature of the biopolymer that should possess chemical groups able to interact with the aroma compound in question. To create interactions, those binding sites should also be available, which is closely linked with the conformational arrangement of the polymer. Besides the polymer, the choice of the solvent and temperature used in the preparation of a film forming solution is of a particular importance because they both affect the solubility and dispersion of the polymeric chains, driving the way they will interact with hydrophobic volatile molecules afterwards. As a result, changes in physicochemical properties of the filmogenic solutions occurred such as change in viscosity whose evolution can be considered as an indicator of interactions. Furthermore, viscosity can also influence drying kinetics and aroma compound retention.

With the aim to compare the ability of different biopolymers to play the role of carrier of an aroma compound when designing an active packaging, four raw materials displaying a contrasted polarity were selected to prepare filmogenic solutions: pectin, AG, SPI, and WG. Regarding the choice of active agents, limonene, carvacrol, and eugenol have been selected for their antimicrobial properties demonstrated in the previous studies (Ben Arfa et al., 2006; González-Estrada et al., 2017) and the wide range of interactions they might create with a given carrier (Table 1). The viscosity of the resulting solutions has been measured and the content in aroma compound of each film has been analyzed in the final material after casting and solvent evaporation (drying).

**Table 1:** Chemical and physical properties of eugenol, carvacrol, and limonene aroma compounds used in the films elaboration.

**Aroma compound**	**M_**W**_ (g/mol)**	**Log P**	**Solubility (g/L at 25^°^C)**	**Polar surface area (Å)**	**Saturated vapor pressure (mmHg at 25^°^C)**
Eugenol	164.2	2.3	2.46	29.4	0.01
Carvacrol	150.2	3.1	1.25	20.2	0.047
Limonene	136.2	4.5	0.012	0	1.54

*Data retrieved from: https://pubchem.ncbi.nlm.nih.gov*.

The filmogenic solutions have been prepared by dispersing the biopolymer at a concentration enabling its complete dispersion in a suitable solvent. Being easy to solubilize in water, SPI was simply dispersed in water under heating (30 min at 50°C) to break the non-covalent bonds (mainly van der Waals interactions) and to favor interaction with water of the polymer chains (Ben Arfa et al., 2007a; Aphibanthammakit et al., 2018). By contrast, WG proteins having a low solubility in water because of a low content of ionized polar amino acids, many hydrophobic interactions between non-polar amino acids and the presence of inter and intra-molecular disulfide bonds stabilizing high molecular weight protein fractions, its dispersion in water required sodium sulfite to reduce disulfide bonds and acetic acid to lower the pH value at 4 (Gastaldi et al., 2007).

For AG and WG, the concentration of biopolymer in both casting solutions was 20% w/w whereas for SPI and pectin the concentration could not exceed 10 and 3% w/w, respectively, to avoid the occurrence of unwanted gelation phenomenon. Moreover, it is also important to check the viscosity of solutions before the addition glycerol as plasticizer because this compound naturally increased the viscosity. As an example, a suspension of AG at 20% w/w was characterized by a weak viscosity (29.1 ± 1.3 mPa.s^−1^) in absence of glycerol and a 2 fold higher value (65 ± 2.3 mPa.s^−1^) in the presence of glycerol at 20% w/w related to AG (*i*.e., 4 g/100 mL). For SPI, the viscosity at 10% w/w was equal to 110 ± 6 mPa.s^−1^ without glycerol and 430 ± 5 mPa.s^−1^ when glycerol was added at 20% w/w related to SPI (*i.e.*, 2 g/100 mL). The effect of glycerol on viscosity clearly depended on the matrix and needed to be evaluated to avoid high viscosity values incompatible with the process of films casting.

Another important parameter to control is the pH of the solution since susceptible to impact aroma compound reactivity. SPI solution had a final pH value around 6.6 without adjustment whereas the final pH of WG solution was adjusted to 4, indicating differences in the net charge of both proteins, and thus different degree of ionization of the chemical groups of the amino acid radicals. Even if water-soluble, AG required to be dispersed in a buffering solution set at pH 5 to limit pH variations that might be induced by the large variability of AG composition. Finally, pectin used for film preparation was high methoxyl pectin with a DE > 50 and the pH value slightly varied around 3.25 depending on the concentration.

Among the aroma compounds tested, limonene is not an ionized compound in contrast to carvacrol and eugenol, which are both characterized by the presence of a hydroxyl group having a pKa equal to 10.42 and 9.94, respectively. Regarding the pH range of the film-forming solutions the properties of selected aroma compounds such as solubility and hydrophobicity should not be affected by their addition in the matrix.

These two last compounds are also identified as hydrogen acceptors or donors (eugenol having two hydrogen bonds acceptors compared to one for carvacrol). This indicated that these compounds would be able to form hydrogen bonds with the different matrices. However, their relative hydrophobic nature (see logP Table 1) also suggested possible interactions with the hydrophobic part of the macromolecules constitutive of the dispersion. It is well known that AG, which is a continuum of arabinogalactan glycoproteins (Lopez-Torrez et al., 2015), and SPI are used for their ability to form and stabilize aroma compound emulsions in relation to their amphiphilic character (Kim and Morr, 1996; Padala et al., 2009).

As already mentioned WG is constituted by proteins characterized by a high content of hydrophobic amino acids, which suggested potential interactions with hydrophobic aroma compounds. Moreover, whatever the treatment applied (thermal *vs*. chemical) to achieve the dispersion of biopolymer in solvent, it can be assumed that the initial polymer structure is changed toward a looser and more open structure resulting in more non-polar sites exposed. Those unmasked sites were expected to become more available for interacting with a given aroma compound that will be introduced subsequently under high shearing to favor its dispersion in the aqueous medium. The sole matrix that could hardly interact with a high hydrophobic compound as limonene was pectin due to its high water soluble polysaccharidic structure (chains of galacturonic acid residues which are partially esterified DE > 50). In this case the addition of an emulsifier (sorbitan monolaurate) was required to favor the entrapment of limonene in the solution but also the film formation.

All the films prepared in the presence of aroma compounds and the different biopolymers were found homogenous suggesting both a suitable dispersion of the polymer in the solvent used, and also of the aroma compound in the filmogenic solution due to the choice of efficient emulsifiers and emulsification process. In contrast with our observations, Fabra et al. (2012) reported a destabilization of the film-forming solutions made of iota-carrageenan and limonene due to creaming during drying process. Finally, Table 2 shows a brief summary of the formulations used to develop the films and/or coated papers including the biopolymer used, aroma compound tested and the use of additives in the matrix (cyclodextrin and/or MMT).

**Table 2:** Formulations of the different films elaborated.

**Material**	**Biopolymer matrix**		**Aroma compound % (w/w db)**			**Presence of additives**	
	**Type**	**Concentration (w/w db)**	**Carvacrol**	**Eugenol**	**Limonene**	**CD**	**MMT**
Coated paper	SPI	10	15	15	5		
	SPI	10	30	30	10		
	SPI	5	6.6	–	–	Yes	
	SPI	5	10			Yes	
	SPI	5	20			Yes	
Film	AG	20	–	–	5		
Film	Pectin	1	–	–	100		
		2	–	–	50		
		3	–	–	33.3		
Film	WG	20	15	–	–		Yes
Coated paper	WG	20	30	–	–		
	WG	20	15				Yes

db, dry based;

AG, acacia gum;

SPI, Soy protein isolate;

WG, wheat gluten;

CD, cyclodextrin;

*MMT, montmorillonites nanoclays*.

#### Effect of the Choice of the Polymer Carrier on Retention

The retention rate will not only depend on the good dispersion or emulsification of the aroma compounds in the matrix of the film-forming solutions but also on the drying step which can be considered as a key step for the retention of aroma compounds as shown in previous studies (Chalier et al., 2007; Kurek et al., 2012). Indeed, for the carvacrol/SPI coated paper, the best conditions to limit the losses were either a fast drying at high temperature (250°C for 20 s using an air dryer) or a slow drying at low temperature (25°C for 3 h at 50 ± 5% RH in an oven). In the present study, the drying conditions applied for all formed films were temperature ≤ 50°C and 50 ± 5% RH.

The retention rate of limonene, the most non-polar and volatile compound was estimated for three matrices AG, SPI, and pectin (Table 3). Retention values in the same range of order were obtained for AG and SPI, whereas the matrix/limonene ratios were greater for SPI than for AG (Table 3). This result could suggest that AG was more efficient carrier to trap limonene than SPI. Moreover, when the limonene content was increased in AG solution, changing the ratio matrix/limonene to 2 and applied as coating on limes, the amount of limonene remaining in the coating made of AG was estimated 2 times higher than the one made of SPI (Konuk et al., 2015). It was not possible to calculate the retention rate and to compare quantitatively the two coatings due to the difficulty of removing the coating. The observed difference could be attributed to high limonene content of the AG filmogenic solution compared to the SPI. This also indicated that AG used at strong concentration (20% w/w) was able to emulsify the limonene even if the ratio matrix/aroma compound was high. It is important to take into account that the emulsification properties of AG are usually exploited for the encapsulation of citrus essential oil (Charve and Reineccius, 2009).

**Table 3:** Influence of formulation of filmogenic solutions made of AG, SPI, and limonene on corresponding retention rate in the dried films.

**Matrix**	**% matrix in solution**	**Ratio matrix/limonene**	**Retention rate of aroma (%)**
AG	20	4	63.2 ± 4.6
SPI	10	20	57.3 ± 4.6
	10	10	67.4 ± 6
Pectin	1	1	0.05
	2	2	18.3
	3	3	31.0

Concerning the SPI, the fact that changing the ratio sparsely modified the retention suggested that it could be decreased without wrong consequences. Using carvacrol instead of limonene, it was previously shown that the retention of aroma compound was lower (70%) for a ratio of 10 as compared to ratio of 30 and 60 (between 82.4 and 88.2%) (Ben Arfa et al., 2007b). This could be explained by the preferential presence of small droplets at the highest concentration and the limited migration of limonene toward the surface.

Regarding pectin films, the limonene retention rates remained relatively weak compared to AG and SPI films. As pectin is characterized by a strong viscosity at low concentration, its dry matter content was limited to 3% w/w in the filmogenic solution. As a result, the amount of limonene related to dry matter was notably high inducing important losses.

The influence of ratio matrix/aroma compound was also clearly evidenced with an increase of the limonene retention from 0.05 to 31% while increasing the ratio from 1 to 3 (Table 3). In this case, the reduction of limonene losses could be explained by a viscosity increase due to the higher dry matter content of the suspension. However, the limonene losses remained important due to the high aroma compound volatility, which was unable to compensate the slowdown of diffusivity through the matrix. Drying is considered as one of the most important steps during the preparation of a biopolymer-based film (Sothornvit et al., 2009). During the drying step, all the volatile agents (including solvents and aroma compounds) are progressively evaporated and this evaporation could be enhanced by the temperature (Kurek et al., 2012). Even if the drying was performed at room temperature in the present study, it had a significant impact explaining the weak retention of the aroma compound.

Filmogenic solutions based on SPI and WG have also been prepared in the presence of carvacrol and eugenol as active agents, which are less volatile and non-polar than limonene. As for SPI solution, the WG solution was characterized by a shear thinning behavior with a flow behavior index *n* of 0.97. However, comparing to SPI, the value for WG was very close to a Newtonian behavior, which might be attributed to the disulfide reduction applied to WG proteins during the preparation of film forming solution. The addition of carvacrol in both filmogenic solutions led to contrasted rheological behavior (Table 4).

**Table 4:** Influence of the formulation of the filmogenic solutions on the consistency (K) and flow behavior index (*n*) measured at 25°C, and the retention rate of the resulting dried films made of SPI, WG, and carvacrol.

**Film formulation**	**Viscosity**		**Retention rate of aroma compound**
	**K (Pa.s)**	***N***	**(%)**
WG^a^	0.03 ± 0.07	0.97	–
WG + 15% carvacrol	6.99 ± 0.11	0.43	76.4 ± 3.2
WG + 30% carvacrol	12.6 ± 0.32	0.41	85.5 ± 17
SPI^b^	0.43 ± 0.001	0.80	–
SPI + 15% carvacrol	0.36 ± 0.016	0.84	73.5 ± 75
SPI + 30% carvacrol	0.43 ± 0.03	0.81	95.3 ± 5.2
SPI + 15% eugenol	0.36 ± 0.08	0.83	ND
SPI + 30% eugenol	0.41 ± 0.02	0.82	75.0 ± 2.5

a *WG solution contained 20% (w/w db), 10% (w/w) glycerol, and 15 or 30% (w/w) carvacrol, as related to dry matter*.

b *SPI solution contained 10% (w/w db), 20% (w/w) glycerol, and 15 or 30% (w/w) caracrol or eugenol as related to dry matter. ND, not determined*.

The viscosity of SPI solution was slightly affected by carvacrol addition resulting in a small decrease at 15% and no change at 30%. It was important to highlight that the effect on viscosity of aroma compound was combined to the glycerol effect, which is specifically pronounced in the case of SPI as mentioned above. It was already described that carvacrol at 30% was able to decrease the consistency of a 10% w/v SPI solution free of glycerol (Ben Arfa et al., 2007a). This change was attributed to the formation of aggregates between soy proteins and carvacrol due to the enhancement of protein-protein interactions at the detriment of protein-water interactions as evidenced by granulometric and microscopic analysis. The heating of the SPI solution prior to the solution preparation was expected to favor this phenomenon by ensuring the partial denaturation of proteins.

The fact that in the presence of glycerol, the effect of carvacrol on consistency was limited, could suggest that the aggregates formation would depend on the glycerol and aroma compound content. Moreover, it is worth noting that the specific structuration of the network resulted to an extreme variability of the retention by SPI. As mentioned above, this lack of repeatability indicated that carvacrol could not be well dispersed in the SPI solution at a concentration as low as 15% due to the formation of clusters heterogeneously distributed in the material. By contrast, when increasing the carvacrol/SPI ratio, a more stable filmogenic solution with smaller clusters seemed to occur as reflected by the increase in viscosity at a higher concentration of carvacrol (30%). This underlined the complexity of the interactions that set up at the interface of the two phases during the preparation of the filmogenic solution. Actually, the presence in the solution of glycerol as plasticizing agent could also play a role in the aroma compound partition phenomenon. As already reported by Kurek et al. (2012), glycerol would act as a competitive agent for carvacrol by occupying places where aroma compound could interact. Such differences could also be explained by a glycerol-induced change in the conformation of the SPI protein toward a more stable and compacted shape less prone to interact with the aroma compound due to a lack of hydrophobic sites available at the protein surface. This assumption was supported by Vagenende et al. (2009) who investigated the mechanisms of protein stabilization and prevention of protein aggregation by glycerol and reported that glycerol-induced protein compaction would be mainly originated from electrostatic interactions that would result in orientations of glycerol molecules at the protein surface such that glycerol would be further excluded. As a result, the use of glycerol would hinder the creation of interactions between carvacrol and SPI and those sites would become less available for interacting and binding carvacrol molecules, which are bigger and less polar molecules than glycerol. However, the resulting of glycerol-proteins, glycerol-carvacrol and carvacrol-protein interactions seems to depend on the carvacrol concentration favoring or not the good repartition and retention of this compound (Table 4).

The addition of eugenol, which is more polar than carvacrol, in the SPI solution led to similar changes in the viscosity whereas the residual amount of eugenol in the final film was lower (1.3 times) than these of carvacrol (Table 4). Considering the fact that carvacrol is less polar than eugenol, a better affinity for the non-polar part of SPI matrix was expected leading to more interactions as reflected by the higher retention rate.

Carvacrol addition from 15 to 30% in WG solution resulted in a decrease of the flow behavior index value (from 0.43 to 0.38) combined with a huge increase of the consistency by a factor 233 for 15% and 420 for 30% carvacrol (Table 4). This indicated that carvacrol addition induced a shear thinning behavior. The change in rheological behavior of WG solutions has already been reported for 15% carvacrol by Mascheroni et al. (2010), who attributed this trend to a good dispersion of the aroma in the solution due to specific interactions between carvacrol and WG protein. As the consistency increased with the carvacrol concentration, it can be assumed that the interactions were amplified. As a result, a better retention was observed when increasing carvacrol concentration from 15 to 30% in WG solution (Table 4).

**Figure 1:**
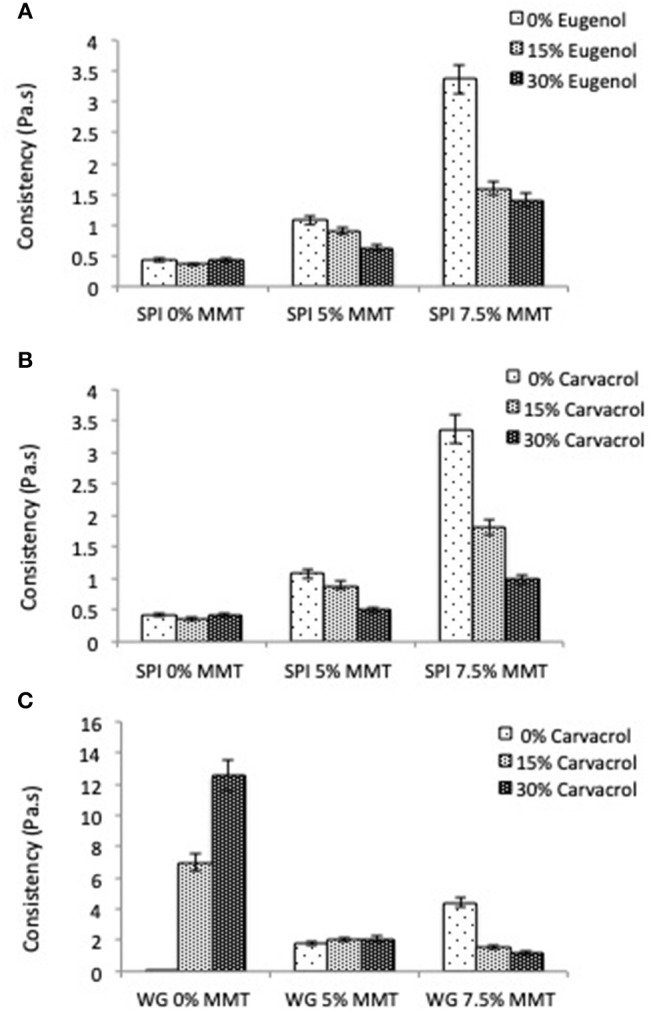
Influence of nanoclays (5 and 7.5%) on the consistency k of filmogenic solutions made of SPI (10% w/w) with **(A)** eugenol or **(B)** carvacrol (15 and 30%) and **(C)** made of WG (20% w/w) with carvacrol.

To compare, replacing WG by SPI led to higher retention (Table 4) when increasing carvacrol concentration from 15 to 30% w/w. This peculiar behavior could be attributed to a better affinity between both components taking into account the good balanced nature of this protein. Hence, it could be proposed that more binding sites of weakly polar nature could be unmasked when increasing the carvacrol/matrix ratio. This would result in a more stable filmogenic solution with smaller clusters as reflected by the small increase in viscosity observed at a higher concentration of carvacrol (Figure 1).

#### Effect of Cyclodextrins on Retention

Carvacrol encapsulated in β-cyclodextrins has been added to the SPI matrix for paper coating in order to better protect carvacrol from lost during process and to increase the potential to control the molecule delivery (Sentze and Fenyvesi, 2018). Cyclodextrins, which are cyclic oligosaccharides with hydrophobic cavity are capable of forming inclusion complexes with varied aroma compounds (Li et al., 2007; Barba et al., 2015). The process of aroma compound inclusion induces the substitution of water for a cavity allowing the insertion of aroma compound as guest. In turn, the release of the guest would be triggered by water substituting the aroma compound in the cavity (Plackett et al., 2007). The content of carvacrol in cyclodextrin is limited since the aroma compound would form CD complex in to 1:1 molar ratio. The carvacrol/CD complexes used in this study were characterized by a carvacrol content of 0.147 g/g of cyclodextrin. This means that the addition of high carvacrol amount in the SPI matrix is needed to reach a sufficient amount of antimicrobial agent. This could lead to a consequent increase of dry matter and viscosity. To limit them, the formulation of the film-forming solution was adapted by decreasing the SPI content from 10 to 5%. Three experiments were performed by varying the carvacrol content and the final dry matter (Table 5). The inclusion of carvacrol in cyclodextrins did not protect it from losses during process. The higher retention was found for the lower amount of carvacrol but the final content of the film was limited (<1.1 g/m^2^). The increase of complexes in the SPI solution to reach 10% of carvacrol relative to dry mater was not beneficial since responsive of a strong increase of carvacrol losses (around 47%). To compare, carvacrol losses remained largely inferior for SPI-coated papers containing 10% of free carvacrol with a value of 30% (Ben Arfa et al., 2007b). It can thus be suggested that a no negligible part of carvacrol was released in the matrix during the process, probably at the stage where it was added to SPI solution due to the presence of water. As a consequence the newly free carvacrol was not well distributed in the coating matrix and was easier lost by volatilization. When carvacrol was simultaneously introduced as free and included in the CD, the losses remained strong but a higher content of residual carvacrol amount was reached in the film. Active compounds are retained in cyclodextrins *via* interactions implying the protons inside the cavity and it was shown that carvacrol strongly interact *via* the protons of the aromatic ring (Barba et al., 2015). However, because of the reversibility of the inclusion process, the introduction of carvacrol/CD complex in a biopolymer solution remains challenging and still needs to be optimized.

**Table 5:** Dry coated weight and carvacrol retention of SPI (5% w/v) papers with added cyclodextrins.

**Carvacrol relative to dry matter (%)**	**Amount of cyclodextrin (%)**	**Final dry matter (%)**	**Dry coated weight (g/m^**2**^)**	**Theoretical carvacrol (g/m^**2**^)**	**Retention %**
6.6	10	15	23.7 ± 0.1	1.56	73.7 ± 7.1
10.5	17	22	24.0 ± 2.1	2.52	53.6 ± 0.5
20.0^*^	17	15	17.3 ± 0.3	3.46	49.1 ± 1.0

* *The final % is due to free carvacrol (50%) and carvacrol-CD (50%)*.

#### Effect of Nanoclays on Retention

To go further in the investigation of the parameters influencing the properties of filmogenic solutions intended for the control delivery of active agents, the effect of introducing nanoclays in their formulation has been studied. To better understand how proteins interact with aroma compound in the presence of nanoclays and how these changes could influence the retention properties, the rheological behavior of filmogenic solutions has been monitored together with X-Ray diffraction analysis. It is worth mention that when nanoclays are introduced in filmogenic solutions, glycerol should also be used to counterbalance the anti-plasticization effect induced by the clay layers on the polymer matrix as already reported by Lavorgna et al. (2010) when developing chitosan based nanocomposite films. In the present study, glycerol was previously mixed with the nanoclays before being introduced in the filmogenic solution. In any event, Kurek et al. (2012) have previously showed that adding glycerol concomitantly to the nanoclays or not in the filmogenic solution would have no impact on the resulting interlayer distance.

As already reported by Mascheroni et al. (2010) and Nassar et al. (2017), the introduction of increasing amounts of nanoclays (from 5 to 7.5% w/w) in filmogenic solutions made of SPI (10% w/w) and WG (20% w/w) led to an increase of the consistency *K* of both solutions with a decrease of the pseudo plastic index *n*. The effect appeared more pronounced in WG solutions due to their rheological behavior nearly Newtonian. This change in the rheological properties of solutions toward a shear thinning behavior was generally attributed to the ability of flow to align the polymers and the highly anisotropic layered silicates in the viscoelastic medium (Krishnamoorti and Yurekli, 2001). The increase of consistency in the presence of MMT could be considered as an indication of a rather good dispersion of nanoclays in the protein matrix possibly leading to intercalation of polymeric chains in the clay interlayer in solution. However, after solvent evaporation and film drying, the complete exfoliation of the nanoclays could not be achieved as evidenced on XRD patterns reported by Mascheroni et al. (2010) and Nassar et al. (2017).

As shown in Figure 1, the addition of increasing amount of eugenol or carvacrol (from 15 to 30% w/w) to filmogenic solutions containing nanoclays (5 and 7.5 % w/w) led to a decrease in consistency *K* with a concomitant small increase of the pseudo plastic index *n* with values varying from 0.66 to 0.75 for SPI with 5% MMT, and from 0.44 to 0.56 for WG with 5% MMT, for instance. This change in the rheological properties was observed whatever the formulation considered, excepted in the case of the WG solution containing 5% MMT where the viscosity remained unchanged (Figure 1C). This might reflect structural rearrangements induced by the addition of aroma compound in the solutions leading to the establishment of new interactions between the different components of the solution. Considering the non-polar nature of both aroma compounds, these latter were expected to interact with non-polar amino acid of the protein chains, which are generally unavailable since buried in the structure in the presence of aqueous solvent. A decrease in viscosity being generally attributed to a lower degree of nanoclays dispersion, this suggested the creation of specific interactions between MMT and aroma compounds that would partially hinder the intercalation of the protein polymeric chains between the clay layers.

**Figure 2:**
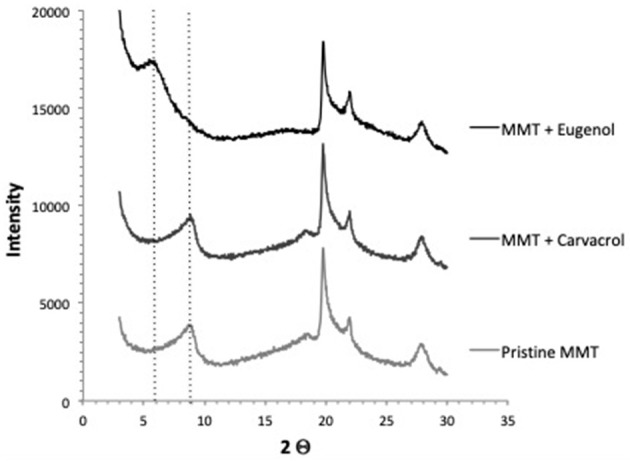
XRD patterns of nanoclays powder (Na^+^-MMT) mixed with eugenol or carvacrol.

With the aim of evaluating the affinity of eugenol and carvacrol for MMT, XRD measurements have been performed on mixtures of MMT with both aroma compounds. Comparison of XRD patterns of nanoclays powder (Na^+^-MMT) and MMT mixed with eugenol showed a shift of the d_001_ peak characteristic of the pristine MMT interlayer distance from 2θ = 8.8 to 5.5° that could be ascribed to an increase of the interlayer space from 10 to 16.1 Å (Figure 2). By contrast, no shift of the d_001_ peak of MMT was observed when nanoclays were mixed with carvacrol whatever the ratios aroma compound/MMT used. This could be interpreted as if eugenol was able to penetrate in the interlayer contrary to carvacrol. Yet, when comparing the molecular dimension of carvacrol and eugenol, which are both small molecules with a low steric hindrance ranging between 6.62 and 10.14 Å, it could be supposed that both molecules could easily enter the gallery and occupy the free spaces available resulting in no change in the MMT interlayer distance. This implies that the shift of the d_001_ peak of MMT observed in the presence of eugenol might rather be due to aggregation of eugenol molecules resulting in the formation of clusters. Such clusters would be prone to interact with nanoclays platelets in the form of aggregated structures entrapping aroma compound as previously evidenced by Ben Arfa et al. (2007a) and Mascheroni et al. (2010) for filmogenic solutions made of SPI or WG.

A better retention of the active compound by MMT was thought to be due to an intercalation of active compound between the clay layers. However, carvacrol and eugenol, which both possess an hydroxyl group, could also be able to establish hydrogen bonds with the hydroxyl groups located at the edges of MMT and the siloxane oxygen atoms of the silicate layers as suggested by other works on pesticide sorption on clays (Lagaly, 2001). This implies that aroma compound could be adsorbed on the edges of nanoclays and also within the gallery but without modifying the interlayer distance. As previously reported by Qi et al. (2016), the soy protein chains can be adsorbed on the surfaces of the interlayer of MMT and at their extremities through hydrogen bonding and electrostatic interaction.

Thus, active molecules could interact with MMT through the establishment of easily reversible interactions involving relatively weak hydrogen bonds on the external surfaces and edges of nanoclays. As demonstrated by Chevillard et al. (2012) such interactions would be a key element for obtaining a suitable delivery system when aiming at developing slow release herbicide. By contrast, the presence of strong interactions would be not suitable for their use as carrier for the controlled release of active agent, due to a too slow release rate and a too large unavailable amount since irreversibly entrapped. This assumption was consistent with XRD patterns previously reported by Mascheroni et al. (2010) and Nassar et al. (2017) for films made of WG or SPI and containing MMT and carvacrol or eugenol. According to these authors, the interlayer distance of the layered silicates was enlarged in the presence of both carvacrol and eugenol, whatever the protein considered but the enlargement appeared too important to be only due to aroma compound. So, it could be proposed that aroma compound addition in the filmogenic solution would facilitate the insertion of protein chains in the clay interlayer by inducing conformational changes. Actually, spontaneous protein unfolding is known to occur when subjected to excessive concentrations of aroma compounds (Semenova et al., 2002).

**Figure 3:**
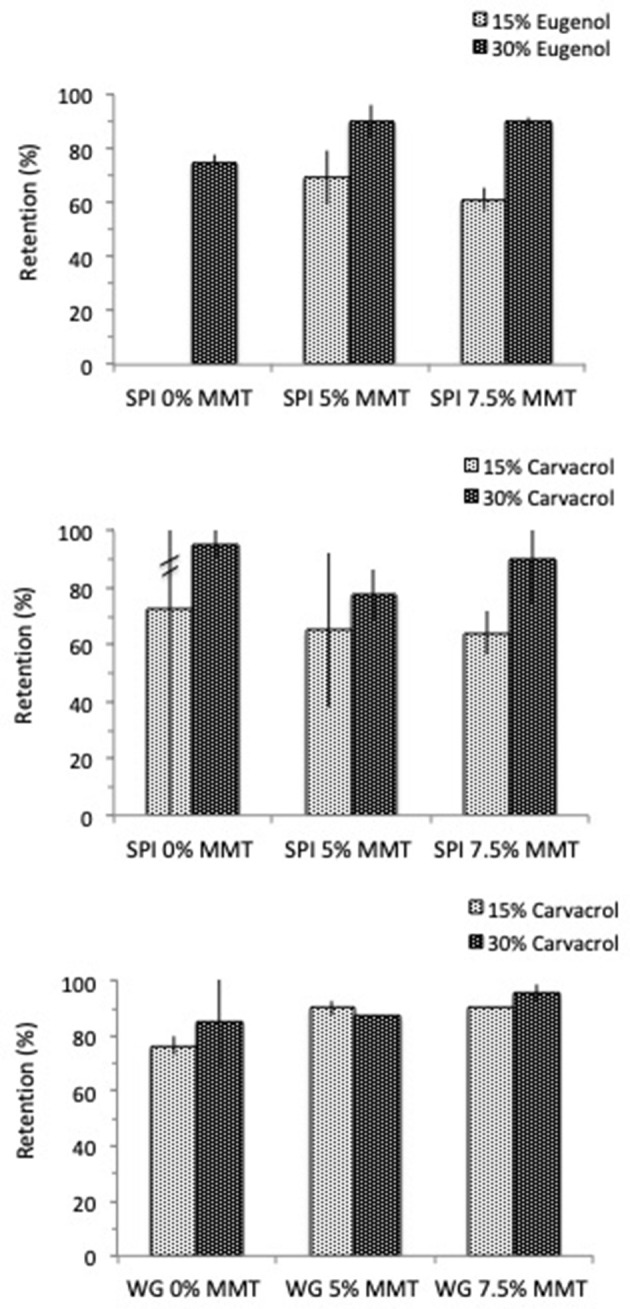
Influence of nanoclays (5 and 7.5%) on the retention of aroma compound by dried films made of SPI (10 wt%) and WG (20 wt%) containing from 15 to 30% eugenol or carvacrol.

Besides the establishment of such specific interactions between MMT, aroma compound and proteins in solution, Figure 3 illustrates how the presence of MMT influenced the retention of aroma in the film after drying.

Introducing MMT in the filmogenic solution led to an improvement of aroma retention in the case of films made of WG and carvacrol, and SPI and eugenol. In contrast, the presence of MMT in SPI film decreased the retention of carvacrol especially in the case where carvacrol was introduced at 15% (w/w db) in the formulation (Figure 3). However, if taking into account the abnormally high standard errors obtained resulting from the extreme variability of the retention rate measured, these results reflected above all highly unstable structures. Actually, this phenomenon was already mentioned above for SPI film forming solution containing 15% carvacrol (Table 5) and was attributed to the formation of clusters heterogeneously distributed in the material. Interestingly, the addition of MMT in the SPI matrix seemed to attenuate this phenomenon to lead to higher retention with less variability but only when MMT were used at 7.5% (instead of 5%) and for a carvacrol concentration of 30% (instead of 15%) (Figure 5). The obtainment of more stable structures and increased carvacrol retention for this specific formulation appeared quite unexpected and would worth being deeper elucidated. Be that as it may, this underlined the complexity of the interactions that set up at the interface of the three components (SPI, carvacrol, and MMT) during the implementation of films from such filmogenic solution.

It was also worth noting that aroma retention by the dried film appeared systematically higher for a concentration of 30% aroma compound than for 15%, excepted in the case of WG films made of carvacrol and 5% MMT for which no significant difference was obtained. Considering that such discrepancy was also observed in films containing or not MMT, it could be supposed that a great part of the aroma compound interact with the proteins, the role played by MMT being mainly to unmask additional protein site by modifying their conformation. Furthermore, all the sites available for interacting and retaining the aroma compound would be probably not occupied meaning that a higher aroma concentration could be used. This assumption was consistent with the formation of aggregated structures in which aroma compounds would be entrapped as clusters by nanoclays platelets through the establishment of specific interactions.

### Influence of the Formulation of Active Films and Coatings on the Release of Aroma Compounds

Controlling the release of antimicrobial compounds from edible films is critical to preserve food products against microbial growth. The release of antimicrobial substances from biopolymers films is dependent on many factors, including interactions between the antimicrobial agent and polymer chains taking place during the manufacturing conditions as previously demonstrated but also structural changes of the polymer matrix induced by the environmental conditions and contact with the food product. Indeed, the release of antimicrobial compounds needs to be avoided during the storage of the material but favored when it is used to pack the food product and more specifically when the risk of microbial growth becomes important. The development of microorganisms is relevant only when the a_w_ of the food product is high enough (>0.7 for fungi and 0.9 for bacteria) and usually it increases with the raise of the temperature. In addition, the entrance of oxygen to the package is not favorable for aerobic microorganisms. Biopolymer coatings or films often exhibit good oxygen barrier properties but poor water barrier properties related to their sensitivity against water. However, this drawback can be advantageously exploited to control the release of antimicrobial agent. Indeed, biopolymers are characterized by glass transition temperatures (*Tg*) values, which can strongly decrease with increasing relative humidity and temperature. The water acts by disrupting hydrogen bonds between the polysaccharides or protein chain segments and it induces the increase of the chain mobility and free volume, which both favor mass transport (Chalier et al., 2009). Water can also compete with aroma compounds for specific interaction sites or when inclusion complexes were added in the polymeric matrix.

**Figure 4:**
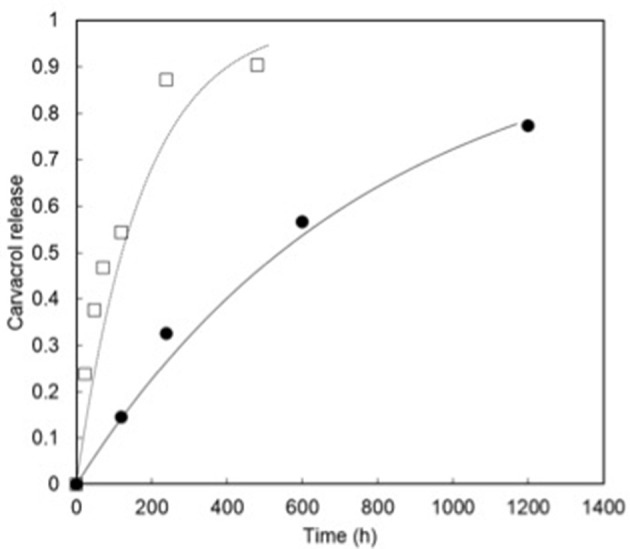
Release of carvacrol from SPI coated paper with (●) or without cyclodextrin (□). The lines correspond to the modeling of the experimental data by Avrami's equation.

This two release behaviors are illustrated in Figure 4, which shows the carvacrol release at 80% RH and 30°C from two SPI-coated papers, one with aroma compound introduced directly in the SPI matrix (free form), and the other containing a mixture of free carvacrol and carvacrol previously included in cyclodextrins (carvacrol-CD complex). As clearly demonstrated in Figure 4 the release slowed down when a part of carvacrol was entrapped in cyclodextrin since 77% of the initial content of carvacrol was delivered after 1,200 h, against 90% after 480 h when the carvacrol was free in SPI-coated paper. As previously demonstrated, high RH and temperature favored the rapid uptake of water vapor by the SPI matrix and led to a decrease of the *Tg* (Chalier et al., 2009). However, in the case of cyclodextrins, carvacrol release was not only dependent on the mobility of soy protein chains constitutive of the matrix but also on the substitution of carvacrol by water in the cavity (Li et al., 2007).

The release of aroma compounds from cyclodextrin is often characterized by Avrami's equation and to quantitatively compare the two kinetics, the equation was applied to both sets of data. This Equation (2) is a mathematical model that originally described a crystallization mechanism derived from the Weibull law.

(2) $$\begin{array}{r} X=1-e^{{-kt}^{n}} \end{array}$$

where *X* = M_t_/M_0_, M_0_ is the total amount of aroma compound contained in the matrix at the beginning of the experiment, M_t_ is the amount of aroma compound retained in the matrix at a given time, *k* is the release constant and *n* is a parameter used to define the transfer mechanism. By taking a double logarithm of both sides of Equation (2), the Equation (3) was obtained with *n* and *k* being easily determined from the interception at ln*t* = 0 and the slope:

(3) $$\begin{array}{r} \ln\left[-\ln\left(1-X\right)\right]=n\ln\;k+n\ln\;t \end{array}$$

The Weibull function allows a good prediction of the entire release curve whereas other models such as Korsmeyer-Peppas (power law) or Higushi matrix model described only the first part of the release (Ritger and Peppas, 1987; Papadopoulou et al., 2006). The Avrami's equation was generally used to describe the release of a drug in a liquid. Indeed, Avrami's equation considers the diffusion of the penetrant into the film, the swelling and the release of drug. Moreover, the exponent *n* is originated from the fact that a depletion zone is gradually created near the boundaries of the release device, and thus, the active agent concentration in the device is not uniform (Kosmidis and Macheras, 2008). Avrami's equation has also been used for the description of the kinetics of active agent released from packaging (Gao et al., 2017; Requena et al., 2017).

The release is considered as a Fickian diffusion phenomenon if *n* lies in the range of 0.54–0.75 (Papadopoulou et al., 2006). When *n* is higher than 0.8 the transfer is described as anomalous combined with pure Fickian diffusion and a case II transport, which reflects the influence of polymer relaxation on the molecule transfer in the matrix. When *n* = 1, the release obeys to the Fick's first law of diffusion and corresponds to a first order reaction which is independent of time, and when *n* > 1 the mechanism is considered as complex (Papadopoulou et al., 2006). Regarding the *n* value, the mechanism of carvacrol release from SPI-coated paper can be considered as diffusive or anomalous suggesting that more than one release mechanism might be involved (Table 6). However, it could be assumed that the diffusive transfer was preponderant since a good prediction was also obtained by modeling the cavacrol release with Fick's second law (Chalier et al., 2009). The fact that the transfer was not strictly diffusive can be explained by the presence of an intermediary zone where the coating penetrated the paper as previously observed (Ben Arfa et al., 2007b).

**Table 6:** n parameter, transfer rate, and correlation coefficient values of the Avrami's equation obtained with packaging based on SPI matrix.

**Matrix**	**Additive**	**Conditions of release**	**Aroma compound and loading (%)**	**Thickness (μm)**	***n***	***k (h^−1^)***	***R^**2**^***
SPI coated paper	–	RH 80%, 30°C	Carvacrol 30	30 ± 50	0.76	41	0.97
	Cyclodextrin	RH 80%, 30°C	Carvacrol 20	58 ± 50	0.95	549	0.93
	–	RH 100%, 30°C	Carvacrol 30	30 ± 5	0.79	5.6	0.93
SPI films	–	RH 100%, 25°C	Carvacrol 30	27 ± 8	0.54	10.1	0.92
	–	RH 100%, 25°C	Carvacrol 30	133 ± 50	0.61	24.6	0.86
	–	RH 100%, 25°C	Eugenol 30	160 ± 30	1.56	2,138	0.96
	5% MMT	RH 100%, 25°C	Carvacrol 30	163 ± 7	0.94	48.0	0.92
	5% MMT	RH 100%, 25°C	Eugenol 30	125 ± 13	1.20	344	0.89

In the presence of carvacrol/CD, the value of *n* was superior to 0.8 showing that the mechanism was not only diffusive. Comparing the release constants *k* between the two papers it was obvious that the presence of carvacrol/CD complex strongly decayed the release (by a factor superior to 10). This means that it could be possible to obtain a more sustainable release of carvacrol by playing on the complex addition in a SPI matrix. Indeed, when studying carvacol release from complex made of aroma compound/CD powder a *n* value of 1.19 and a *k* value of 3,393 h^−1^ were obtained, which was in agreement with a complex mechanism and suggested that increasing the complex amount could further slowdown the release.

Another mean to control the release was to modify the relative humidity as previously stated (Chalier et al., 2009) and to adapt it to the storage conditions of the product. This change led to a significant impact on the *k* value when modeling the Avrami's equation since a lower value was obtained when the kinetic of carvacrol from SPI-coated paper was conducted at 100% RH compared to 80%. As shown in Table 6, the *k* value decreased by 80 times, whereas when the release was characterized by the apparent diffusivity, the difference between the two RH was found less pronounced (15 times weaker for a RH of 80% than for 100%, Chalier et al., 2009). So, it could be suggested that in contrast to the apparent diffusivity, *k* takes into account not only the transfer of the active compound, but also the change of polymer matrix and the nature of the mechanism involved. Indeed k is strongly dependent on the values of *n*.

It is also possible to vary the thickness of the film to control the release as shown in Table 6. When the thickness of a SPI films was increased (from 27 to 133 μm), the release rate of carvacrol decreased two times (*k* = 10.1 and 24.6 h^−1^, respectively). It is generally described that film thickness exerts a direct influence on the mentioned parameters (Lu et al., 2016; Moghadam and Voorhees, 2016). Decreasing film thickness also induced important losses of volatile aroma compound during the film processing due to a greater interface exposed to air Hence, increasing film thickness had the double benefit to induce higher amount of active agent and to lead to a more efficient release. Comparison between coated paper and films showed significant differences in the values of *n* and *k* in spite of similar thicknesses (≈30 μm) (Table 6). Notably, results led to conclude that the transfer of carvacrol in the film was significantly more diffusive and slower than in the coated paper. Even if such difference in release could be due to difference in temperature of the test, which was 5 degree more in the case of coating paper, it also confirmed the role played by the intermediary zone where the coating penetrated the paper, which clearly affected the transfer by increasing it.

The nature of the aroma compound is another parameter that could affect the release since prone to result in new interactions with the polymer matrix due to its specific physicochemical properties. To illustrate this assumption, it was demonstrated above that carvacrol was better retained by SPI than eugenol (Figure 3). Thus, replacing carvacrol by eugenol would caused a change in the mechanism of release, which was not only diffusive but also became complex. Moreover, the release of eugenol was very slow with a *k* value of almost 100 times higher than these obtained with carvacrol (Table 6). This was in agreement with the weak volatility of eugenol and also the slightly higher film thickness (160 μm against 133 μm for eugenol and carvacrol, respectively). Moreover, it chiefly suggested the presence of a strong interaction between eugenol and SPI, which might be in contradiction with retention results if the role played by water is not taken into account. Thus, as eugenol is a relatively polar compound, a competition with water for the same site of bonding on the protein could be evocated. Furthermore, the comparison of the *Tg* values obtained in the present study for the films maintained at 50 % RH showed that eugenol addition in the SPI induced a decrease of *Tg* from 42 ± 1.6 to 36.6 ± 2°C corresponding to a plasticization effect. In contrast, the addition of carvacrol to SPI provoked an increase of *Tg* (55.6°C) *i.e*., reflecting an anti-plasticization effect. It is important to highlight that at higher RH values, the triggering effect of water would be limited for the eugenol/SPI films compared to the carvacrol/SPI films.

The presence of MMT could also affect the release of aroma compounds, the transfer being in that case not only diffusive as indicated in Table 6. For eugenol, the release was increased as demonstrated by the decrease of *k* value (6 times) while for carvacrol, the value of *k* increased with a factor of 4. This contrasted behavior could be explained by the specific interactions involved for each aroma compound with the MMT and the protein network as described above.

The nature of the matrix in which the active agent is trapped could also influence the release mechanism. For comparison purposes, the release of carvacrol through WG film and WG-coated paper was monitored at high relative humidity (100% RH) and 25°C and then modeled by Avrami's equation (Table 6). Comparison with the carvacrol release from SPI films having close thickness indicated that the impact of the matrix was limited since for both case, the transfer was controlled by diffusive mechanism and the *k* values were similar (Table 7). Moreover, the addition up to 7.5% of MMT in the film matrix caused a slight decrease of the carvacrol release as evidenced by the increase of *k* value. However, the increase of MMT at a rate of 10% induced an increase of the transfer. Similar behavior was reported for paper coated with gluten, but the change in diffusivity was observed for an addition of 7% of MMT (Mascheroni et al., 2011). The data treatment with Avrami's equation confirmed that the transfer of carvacrol was diffusive and was affected by the MMT content with a decrease of *k* at 7% (Table 7).

**Table 7:** Carvacrol's *n* parameter, rate transfer, and correlation coefficient values of the linearization of the Avrami's equation for WG films and WG coated paper.

**Matrix**	**% MMT**	***n***	***k* (h^−1^)**	***R^2^***
Wheat gluten film	0	0.702	24.2	0.99
	5	0.749	33.2	0.97
	7.5	0.790	36.9	0.95
	10	0.669	17.0	0.90
Wheat gluten coated paper	0	0.704	28.7	0.96
	5	0.774	35.5	0.92
	7	0.678	20.6	0.98

Dry coated weight and carvacrol retention of SPI (5% w/v) papers with added cyclodextrins.

The close values of thickness (~200 μm) between the films and the coated papers and similar formulation (glycerol being added in both cases) led to expect a similar *n* parameter and transfer rate. However, the observed difference with varying MMT could be explained by a final concentration in the WG layer of the paper superior to those targeted due to a partial penetration of WG in the paper. By consequence, if the final aroma concentration was closer to 10% than 7%, the behavior of the two materials will be very similar.

**Figure 5:**
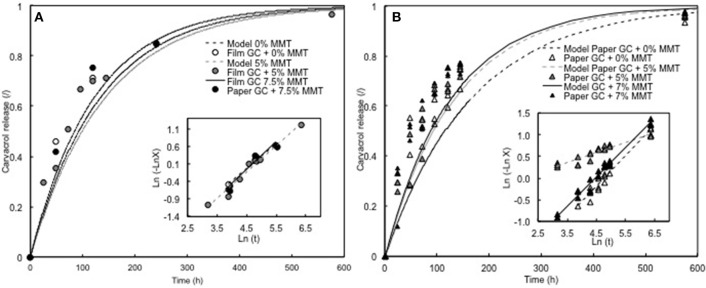
Release of carvacrol and linearization of Avrami's model to WG films with Carvacrol + MMT **(A)** and WG-coated papers with Carvacrol + MMT **(B)**.

As shown in Tables 5, 6, the model of Avrami fitted approximately through time the release of aroma compounds from each specific matrix (film or coated paper) with values of Pearson coefficient correlation when plotting ln(-ln *X*) against ln*t* varying between 0.86 and 0.99. Notwithstanding a good correlation coefficient (Table 7), experimental data could be off the proposed model in the release curves. This was evidenced on the modeling of WG-coated paper (Figure 5B). A reason why experimental data did not overlapped the model could be explained by the fact that the aroma compounds were not homogeneously distributed in the matrix but entrapped in large aggregated structures as explained above and described by Mascheroni et al. (2010, 2011). As a consequence, high amounts of aroma compound were preferentially delivered at the beginning of the storage experiment.

## Conclusion

The retention and release of aroma compounds from biopolymer matrices (films, coated paper) is complex to predict and several factors need to be taken into account despite a rather good knowledge of intrinsic physico-chemical properties of biopolymer and aroma compound. In this study the nature of the carrier matrix (WG, SPI), combined or not with the addition of cyclodextrin and MMT, have been shown to exert a strong influence on the retention and release of carvacrol, eugenol and limonene. However, a general behavior cannot be deduced since each aroma compound interacts specifically with a given matrix, and the resulting interactions not only affect the retention and release rate but also the mechanism of transfer. Furthermore, introducing MMT or cyclodextrins in the formulation have been shown to lead to competition phenomenon driving new interactions and structures that offer a wide range of release behaviors. Avrami's equation was successful used to model the release allowing to characterize the transfer for each system and to evidence that the mechanism is not always diffusive-controlled.

This study underlined that obtaining a good homogeneity of aroma compounds through the carrier matrix as well as implementing strong interactions between both components could be tricky to achieve, but they were not restraining factors for obtaining an efficient delivery system. Actually, a high retention could be reached independently of the release pattern, which in turn could fast or slow depending of the intended target in terms of type of food, storage conditions, storage life, microbial contamination, etc. Thus, using biopolymers as carriers for aroma compound possessing active properties allow obtaining a large panel of tunable active packaging that could be easily adapted to product requirements by only changing some parameters. Considering the weak mechanical properties of films made of biopolymers, it could be envisaged to apply them as a coating directly on the product or on a pare sheet or to introduce them as an active label in the package where the delivery of active agent could occur in the head space. Lastly, in the current environmental context, the bio-based nature of all the components entering in the formulation of such packaging could also be considered as a relevant added value.

Beyond these statements, the major question remaining when designing an active packaging is how to predict its efficiency against a targeted microorganism. To face this key issue it is important to estimate when and how amount of the active compound need to be released to control the rate of growth. The usual approach is to test the packaging *in vivo* after inoculation of the product by the targeted microorganism and storage in usual conditions or in conditions favoring its development. The other approach consists to predict the antimicrobial activity of the packaging by considering both content of aroma compound in the packaging and transfer parameters obtained by modeling the release and taking into account the minimal inhibition concentration against targeted microorganism. These two approaches are time-consuming and require a good knowledge of the growth rate of the microorganism. For these reasons, it appears essential to develop a new methodology allowing in the same time to monitor the microorganism development and to quantify the active agent release.

## Author Contributions

PC and EG directed and designed the different studies. JW-R and TR analyzed and interpreted the data. JW-R, PC, and EG drafted the manuscript and TR provided a critical review of it.

### Conflict of Interest Statement

The authors declare that the research was conducted in the absence of any commercial or financial relationships that could be construed as a potential conflict of interest.

## References

[B1] Ali A. Maqbool M. Ramachandran S. Alderson P. G. (2010). Gum arabic as a novel edible coating for enhancing shelf-life and improving postharvest quality of tomato (*Solanum lycopersicum* L.) fruit. Postharvest Biol. Technol. 58, 42–47. 10.1016/j.postharvbio.2010.05.005

[B2] Alma M. H. Ertaş M. Nitz S. Kollmannsberger H. (2007). Chemical composition and content of essential oil from the bud of cultivated Turkish clove (*Syzygium aromaticum* L.). BioResources 2, 265–269.

[B3] Alves-Silva J. M. Dias dos Santos S. M. Pintado M. E. Pérez-álvarez J. A. Fernández-López J. Viuda-Martos M. (2013). Chemical composition and *in vitro* antimicrobial, antifungal and antioxidant properties of essential oils obtained from some herbs widely used in Portugal. Food Control 32, 371–378. 10.1016/j.foodcont.2012.12.022

[B4] Antosik A. K. Wilpiszewska K. Wróblewska A. Markowska-Szczupak A. Malko M. W. (2017). Fragrant starch-based films with limonene. Curr. Chem. Lett. 6, 41–48. 10.5267/j.ccl.2017.2.002

[B5] Aphibanthammakit C. Nigen M. Gaucel S. Sanchez C. Chalier P. (2018). Surface properties of *Acacia senegal* vs *Acacia seyal* films and impact on specific functionalities. Food Hydrocoll. 82, 519–533. 10.1016/j.foodhyd.2018.04.032

[B6] Balaguer M. P. Borne M. Chalier P. Gontard N. Morel M.-H. Peyron S.. (2013). Retention and release of cinnamaldehyde from wheat protein matrices. Biomacromolecules 14, 1493–1502. 10.1021/bm400158t 23570552

[B7] Baldwin E. A. Hagenmaier R. Bai J. (2011). Edible Coatings and Films to Improve Food Quality. Boca Raton, FL: CRC Press.

[B8] Barba C. Eguinoa A. Maté J.I. (2015). Preperationa and characterisation of b-cyclodextrin inclusion complexes as a tool of a controlled antimicrobial release in Whey protein edible films. LWT Food Sci. Technol. 64, 1362–1369. 10.1016/j.lwt.2015.07.060

[B9] Ben Arfa A. Chrakabandhu Y. Chalier P. Preziosi-Belloy L. Gontard N. (2007a). Coating papers with soy protein isolates as inclusion matrix of carvacrol. Food Res. Int. 40, 22–32. 10.1016/j.foodres.2006.07.011

[B10] Ben Arfa A. Combes S. Preziosi-Belloy L. Gontard N. Chalier P. (2006). Antimicrobial activity of carvacrol related to its chemical structure. Lett. Appl. Microbiol. 43, 149–154. 10.1111/j.1472-765X.2006.01938.x 16869897

[B11] Ben Arfa A. Preziosi-Belloy L. Chalier P. Gontard N. (2007b). Antimicrobial paper based on a soy protein isolate or modified starch coating including carvacrol and cinnamaldehyde. J. Agric. Food Chem. 55, 2155–2162. 10.1021/jf0626009 17305355

[B12] Campos C. A. Gerschenson L. Flores S. K. (2011). Development of edible films and coatings with antimicrobial activity. Food Bioprocess Technol. 4, 849–875. 10.1007/s11947-010-0434-1

[B13] Chalier P. Ben Arfa A. Guillard V. Gontard N. (2009). Moisture and temperature triggered release of a volatile active agent from soy protein coated paper: effect of glass transition phenomena on Carvacrol diffusion coefficient. J. Agric. Food Chem. 57, 658–665. 10.1021/jf802254p 19154166

[B14] Chalier P. Ben Arfa A. Preziosi-Belloy L. Gontard N. (2007). Carvacrol losses from soy protein coated papers as a function of drying paper conditions J. Appl. Polym. Sci. 106, 611–620. 10.1002/app.26662

[B15] Charve J. Reineccius G. A. (2009). Encapsulation performance of proteins and traditional materials for spray dried flavors. J. Agric. Food Chem. 57, 2486–2492. 10.1021/jf803365t 19231860

[B16] Chevillard A. Angellier-Coussy H. Guillard V. Gontard N. Gastaldi E. (2012). Controlling pesticide release via structuring agropolymer and nanoclays based materials. J. Hazard. Mater. 205–206, 32–39. 10.1016/j.jhazmat.2011.11.093 22230752

[B17] Devi K. P. Nisha S. A. Sakthivel R. Pandian S. K. (2010). Eugenol (an essential oil of clove) acts as an antibacterial agent against *Salmonella typhi* by disrupting the cellular membrane. J. Ethnopharmacol. 130, 107–115. 10.1016/j.jep.2010.04.025 20435121

[B18] Di Pasqua R. Betts G. Hoskins N. Edwards M. Ercolini D. Mauriello G. (2007). Membrane toxicity of antimicrobial compounds from essential oils. J. Agric. Food Chem. 55, 4863–4870. 10.1021/jf0636465 17497876

[B19] Efrati R. Natan M. Pelah A. Haberer A. Banin E. Dotan A. (2014). The combined effect of additives and processing on the thermal stability and controlled release of essential oils in antimicrobial films. J. Appl. Polym. Sci. 131:40564 10.1002/app.40564

[B20] Fabra M. J. Chambin O. Voilley A. Gay J.-P. Debeaufort F. (2012). Influence of temperature and NaCl on the release in aqueous liquid media of aroma compounds encapsulated in edible films. J. Food Eng. 113:360 10.1016/j.jfoodeng.2012.06.002

[B21] Gao H. Fang X. Chen H. Qin Y. Xu F. Jin T. Z. (2017). Physiochemical properties and food application of antimicrobial PLA film. Food Control 73, 1522–1531. 10.1016/j.foodcont.2016.11.017

[B22] Gastaldi E. Chalier P. Guillemin A. Gontard N. (2007). Microstructure of protein-coated paper as affected by physico-chemical properties of coating solutions. Colloids Surf. A 301, 301–310. 10.1016/j.colsurfa.2006.12.079

[B23] Gennadios A. Brandenburg A. H. Park J. W. Weller C. L. Testin R. F. (1994). Water vapor permeability of wheat gluten and soy protein isolate films. Ind. Crops Prod. 2, 189–195. 10.1016/0926-6690(94)90035-3

[B24] González-Estrada R. R. Calderón-Santoyo M. Ragazzo-Sánchez A. Peyron S. Chalier P. (2017). Antimicrobial soy protein isolate-based films: physical characterisation, active agent retention and antifungal properties against *Penicillium italicum*. Int. J. Food Sci. Technol. 53, 921–929. 10.1111/ijfs.13664

[B25] Han J. H. (ed.). (2005). Antimicrobial packagings, in Innovations in Food Packaging, Food Sciences and Technology: International Series (Winnipeg, MB: Academic Press), 80–107.

[B26] Kim Y. D. Morr C. V. (1996). Microencapsulation properties of gum Arabic and several food proteins: spray-dried orange oil emulsion particles. J. Agri. Food Chem. 44, 1314–1320. 10.1021/jf9503927

[B27] Konuk D. Rasetti L. González-Estrada R. Calderón-Santoyo M. Ragazzo-Sanchez J. A. Korel F. (2015). Impact of novel bioactive edible coatings enriched with limonene on the postharvest quality of limes, in 29th EFFoST International Conference Proceedings 10-12 November 2015 (Athens).

[B28] Kosmidis K. Macheras P. (2008). Monte Carlo simulations of drug release from matrices with periodic layers of high and low diffusivity. Int. J. Pharm. 354, 111–116. 10.1016/j.ijpharm.2007.10.036 18063328

[B29] Krishnamoorti R. Yurekli K. (2001). Rheology of polymer layered silicate nanocomposites. Curr. Opin. Colloid Interface Sci. 6, 464–470. 10.1016/S1359-0294(01)00121-2

[B30] Kurek M. Descours E. Galic K. Voilley A. Debeaufort F. (2012). How composition and process parameters affect volatile active compounds in biopolymer films. Carbohydr. Polym. 88, 646–656. 10.1016/j.carbpol.2012.01.012

[B31] Labuza T. P. Breene W. M. (1989). Applications of “active packaging” for improvement of shelf-life and nutritional quality of fresh and extended shelf-life foods. J. Food Process. Preserv. 13, 1–69. 10.1159/000416709 2658962

[B32] Lagaly G. (2001). Pesticide–clay interactions and formulation. Appl. Clay Sci. 18, 205–209. 10.1016/S0169-1317(01)00043-6

[B33] Lavorgna M. Pischelli F. Mangiacapra P. Buonocuore G. (2010). Study of the combined effect of both clay and glycerol plasticizer on the properties of chitosan films. Carbohydr. Polym. 82, 291–298. 10.1016/j.carbpol.2010.04.054

[B34] Li X. Jin Z. Wang J. (2007). Complexation of allyl isothiocyanate by α- and β-cyclodextrin and its controlled release characteristics. Food Chem. 103, 461–466. 10.1016/j.foodchem.2006.08.017

[B35] López P. Sánchez C. Batlle R. Nerín C. (2007). Development of flexible antimicrobial films using essential oils as active agents. J. Agric. Food Chem. 55, 8814–8824. 10.1021/jf071737b 17880148

[B36] Lopez-Torrez L. Nigen M. Williams P. Doco T. Sanchez C. (2015). *Acacia senegal* vs. *Acacia seyal* gums - Part 1: Composition and structure of hyperbranched plant exudates. Food Hydrocolloids 51, 41–53. 10.1016/j.foodhyd.2015.04.019

[B37] Lu F. Yu H. Yan C. Yao J. (2016). Polulactic acid nanocomposite films with spherical nanocelluloses as efficient nucleation agents: effects on crystallization, mechanical and thermal properties. RSC Adv. 6, 46008–46018. 10.1039/C6RA02768G

[B38] Madene A. Jacquot M. Sher J. Desobry S. (2006). Flavour encapsulation, and controlled release-a review. Int. J. Food Sci. Technol. 41, 1–21. 10.1111/j.1365-2621.2005.00980.x

[B39] Malhotra B. Keshwani A. Kharkwal H. (2015). Antimicrobial food packaging potential and pitfalls. Front. Microbiol. 6:611. 10.3389/fmicb.2015.00611 26136740 PMC4468856

[B40] Mascheroni E. Chalier P. Gontard N. Gastaldi E. (2010). Designing of a wheat gluten/montmorillonite based system as carvacrol carrier: rheological and structural properties. Food Hydrocolloids 24, 406–413. 10.1016/j.foodhyd.2009.11.007

[B41] Mascheroni E. Guillard V. Gastaldi E. Gontard N. Chalier P. (2011). Anti-microbial effectiveness of relative humidity-controlled carvacrol release from wheat gluten/montmorillonite coated papers. Food Control 22, 1582–1591. 10.1016/j.foodcont.2011.03.014

[B42] Mastromatteo M. Mastromatteo M. Conte A. Del Nobile M. A. (2010). Advances in controlled release devices for food packaging applications. Trends Food Sci. Technol. 21, 591–598. 10.1016/j.tifs.2010.07.010

[B43] Mkaddem M. Bouajila J. Ennajar M. Lebrihi A. Mathieu F. Romdhane M. (2009). Chemical composition and antimicrobial and antioxidant activities of mentha (longifolia L. and viridis) essential oils. J. Food Sci. 74, M358–M363. 10.1111/j.1750-3841.2009.01272.x 19895481

[B44] Moghadam M. M. Voorhees P. W. (2016). Thin film phase transformation kinetics: from theory to experiment. Scr. Mater. 124, 164–168. 10.1016/j.scriptamat.2016.07.010

[B45] Nassar S. F. Dombre C. Gastaldi E. Touchaleaume F. Chalier P. (2017). Soy protein isolate nanocomposite film enriched with eugenol, an antimicrobial agent: interactions and properties. J. Appl. Polym. Sci. 135, 45941–45950. 10.1002/app.45941

[B46] Padala S. R. Williams P. A. Shashi R Williams P. A. Phillips G. O. (2009). Adsorption of gum arabic, egg white protein, and their mixtures at the oil-water interface in limonene oil-in-water emulsions. J. Agric. Food Chem. 57, 4964–4973. 10.1021/jf803794n 19422219

[B47] Papadopoulou V. Kosmidis K. Vlachou M. Macheras P. (2006). On the use of the Weibull function for the discernment of drug release mechanisms. Int. J. Pharm. 309, 44–50. 10.1016/j.ijpharm.2005.10.044 16376033

[B48] Pavlath A. E. Voisin A. Robertson G. H. (1999). Pectin-based biodegradable water insoluble films. Macromol. Symp. 140, 107–113. 10.1002/masy.19991400112

[B49] Pérez Espitia P. J. Du W. Avena-Bustillos R. Ferreiras soares N. McHugh T. H. (2014). Edible films from pectin: physical-mechanical and antimicrobial properties - A review. Food Hydroolloids 35, 287–296. 10.1016/j.foodhyd.2013.06.005

[B50] Perisco P. Abrogi V. Carfagna C. Cerruti P. Ferrocino I. Mauriello G. (2009). Nanocomposite polymer films containing carvacrol for antimicrobial active packaging. Polym. Eng. Sci. 49, 1447–1455. 10.1002/pen.21191

[B51] Plackett D. Ghanbari-Siahkali A. Szente L. (2007). Behavior of α- and β-cyclodextrin-encapsulated allyl isothiocyanate as slow-release additives in polylactide-co-polycaprolactone films. J. Appl. Polymer Sci. 105, 2850–2857. 10.1002/app.26344

[B52] Qi G. Wang L. N. Sun X. S. (2016). Development of high-strength soy protein adhesives modified with sodium montmorillonite clay. J. Am. Oil Chem. Soc. 93, 1509–1517. 10.1007/s11746-016-2890-x

[B53] Ramos M. Jiménez A. Peltzer M. Garrigós M. C. (2012). Characterization and antimicrobial activity studies of polypropylene films with carvacrol and thymol for active packaging. J. Food Eng. 109, 513–519. 10.1016/j.jfoodeng.2011.10.031

[B54] Reineccius G. A. (1989). Flavour encapsulation. Food Rev Int. 5:448 10.1080/87559128909540848

[B55] Requena R. Vargas M. Chiralt A. (2017). Release kinetics of carvacrol and eugenol from poly(hydroxybutyrate-co-hydroxyvalerate) (PHBV) films for food packaging applications. Eur. Polym. J. 92, 185–193. 10.1016/j.eurpolymj.2017.05.008

[B56] Ribeiro-Santos R. Andrade M. Ramos de Melo N. Sanches S. A. (2016). Use of essential oils in active food packaging: recent advances and future trends. Trends Food Sci. Technol. 61, 132–140. 10.1016/j.tifs.2016.11.021

[B57] Ritger P. L. Peppas N. A. (1987). A simple equation for description of solute release I. Fickian and non-fickian release from non-swellable devices in the form of slabs, spheres, cylinders or discs. J. Control. Release 5, 23–36. 10.1016/0168-3659(87)90034-4 25356469

[B58] Rivera J. Crandall P. G. Bryan C. A. O. Ricke S. C. (2015). Essential oils as antimicrobials in food systems - A review. Food Control 54, 111–119. 10.1016/j.foodcont.2014.12.040

[B59] Rodríguez-Lafuente A. Batlle R. Nerín C. (2007). The use of natural essential oils as antimicrobial solutions in paper packaging. Part II. Prog. Org. Coat. 60, 33–38. 10.1016/j.porgcoat.2007.06.006

[B60] Semenova M. G. Antipova A. S. Wassermab L. A. Misharina T. A. Golovnya R. V. (2002). Binding of aroma compounds with legumin. II. Effect of hexyl acetate on thermodynamic properties of 11S globulin in aqueous medium. Food Hydrocolloids 16, 565–571. 10.1016/S0268-005X(02)00018-8

[B61] Sentze L. Fenyvesi É. (2018). Cyclodextrin-enabled polymer composites for packaging. Molecules 23:1556 10.3390/molecules23071556 PMC6100494 29954121

[B62] Sothornvit R. Rhim J. W. Hong S. J. (2009). Effect of nano-clay type on the physical and antimicrobial properties of whey protein isolate/clay composite films. J. Food Eng. 91, 468–474. 10.1016/j.jfoodeng.2008.09.026

[B63] Vagenende V. Yap M. G. Trout B. L. (2009). Mechanisms of protein stabilization and prevention of protein aggregation by glycerol. Biochemistry 48, 11084–11096. 10.1021/bi900649t 19817484

[B64] Vermeiren L. Devlieghere F. van Beest M. Debevere J. (1999). Developments in the active packaging of foods. Trends Food Sci. Technol. 10,77–86. 10.1016/S0924-2244(99)00032-1

